# Novel Mutant Alleles Reveal a Role of the Extra-Large G Protein in Rice Grain Filling, Panicle Architecture, Plant Growth, and Disease Resistance

**DOI:** 10.3389/fpls.2021.782960

**Published:** 2022-01-03

**Authors:** Akshaya K. Biswal, Ting-Ying Wu, Daisuke Urano, Rémi Pelissier, Jean-Benoit Morel, Alan M. Jones, Ajaya K. Biswal

**Affiliations:** ^1^Department of Biology, University of North Carolina at Chapel Hill, Chapel Hill, NC, United States; ^2^International Maize and Wheat Improvement Center (CIMMYT), Texcoco, Mexico; ^3^Temasek Life Sciences Laboratory, Singapore, Singapore; ^4^Department of Biological Sciences, National University of Singapore, Singapore, Singapore; ^5^PHIM, CEFE, Institut Agro, INRAE, CIRAD, Université de Montpellier, Montpellier, France; ^6^PHIM, INRAE, CIRAD, Institut Agro, Université de Montpellier, Montpellier, France; ^7^Department of Pharmacology, University of North Carolina at Chapel Hill, Chapel Hill, NC, United States; ^8^Department of Biochemistry and Molecular Biology, University of Georgia, Athens, GA, United States; ^9^Complex Carbohydrate Research Center, University of Georgia, Athens, GA, United States

**Keywords:** rice, heterotrimeric G proteins, RGA1, extra-large G proteins (XLGs), CRISPR/Cas9, OsXLG

## Abstract

Plant growth and grain filling are the key agronomical traits for grain weight and yield of rice. The continuous improvement in rice yield is required for a future sustainable global economy and food security. The heterotrimeric G protein complex containing a canonical α subunit (RGA1) couples extracellular signals perceived by receptors to modulate cell function including plant development and grain weight. We hypothesized that, besides RGA1, three atypical, extra-large GTP-binding protein (XLG) subunits also regulate panicle architecture, plant growth, development, grain weight, and disease resistance. Here, we identified a role of XLGs in agronomic traits and stress tolerance by genetically ablating all three rice XLGs individually and in combination using the CRISPR/Cas9 genome editing in rice. For this study, eight (three single, two double, and three triple) null mutants were selected. Three XLG proteins combinatorically regulate seed filling, because loss confers a decrease in grain weight from 14% with loss of one XLG and loss of three to 32% decrease in grain weight. Null mutations in *XLG2* and *XLG4* increase grain size. The mutants showed significantly reduced panicle length and number per plant including lesser number of grains per panicle compared to the controls. Loss-of-function of all individual XLGs contributed to 9% more aerial biomass compared to wild type (WT). The double mutant showed improved salinity tolerance. Moreover, loss of the *XLG* gene family confers hypersensitivity to pathogens. Our findings suggest that the non-canonical XLGs play important roles in regulating rice plant growth, grain filling, panicle phenotype, stress tolerance, and disease resistance. Genetic manipulation of *XLG*s has the potential to improve agronomic properties in rice.

## Introduction

Grain yield has been increased substantially over the past 50 years; however, a continuous innovation is needed to increase rice production by ∼ 50% from the current level to feed the fast-growing global population by 2050 (Ray et al., 2013). The size of the grain is a prime breeding target for the introduction of new breeding rice varieties, as it affects weight, grain yield, and quality of the grain (Wang et al., 2012; Sun et al., 2018). To unravel the molecular basis of breeding for higher yield rice production, the identification of allelic differences in other genes related to grain yield is essential.

Heterotrimeric GTP-binding protein (G protein) complexes regulate numerous cellular functions in higher plant cells (Supplementary Table 1). The canonical plant G protein complex constitutes a guanine nucleotide-binding Gα subunit with GTPase activity, an obligate Gβγ dimer, and a seven-transmembrane regulator of G signaling (RGS) protein that modulates activation of Gα (Jones et al., 2011). The inactive (GDP-bound) Gα forms a heterotrimer with Gβγ. The activated GTP-bound Gα dissociates from the heterotrimer, and the Gα subunit and the Gβγ dimer target corresponding effectors to initiate the cascades of responses to the external stimuli. The Gα subunit hydrolyzes the GTP-bound to GDP with its intrinsic GTPase activity and returns to inactive stage, thereby completing the GTPase cycle and consequently stopping all signals. In addition to the canonical Gα subunit, plants possess atypical extra-large GTP-binding proteins (XLG) (Assmann, 2002; Jones, 2002; Maruta et al., 2015; Urano et al., 2016). XLG proteins have a Gα-like domain in the C-terminal half while the N-terminal half has a putative nuclear localization signal and a cysteine-rich region whose function or mode of action is unknown. XLG proteins lack structural requisites for nucleotide binding (Temple and Jones, 2007), and indeed binding is barely detectable for Arabidopsis XLG (Lou et al., 2019). Arabidopsis XLGs interact with AGB1 and AtRGS1 in a nucleotide-independent manner (Lou et al., 2019).

The rice genome encodes one canonical Gα (Ishikawa et al., 1995), one Gβ (Ishikawa et al., 1996), and five Gγ subunits (Kato et al., 2004; Chakravorty et al., 2011; Botella, 2012). Iwasaki et al. (2008) reported four XLG genes in rice. However, the encoded XLG3 protein is relatively small and lacked the characteristic N-terminal domain and other critical structure to consider it as a member of this family. Mutations in rice G protein genes are associated with major agronomical traits such as dwarfness, panicle formation, and grain size (Fujisawa et al., 1999; Mao et al., 2010; Sun et al., 2018). Two non-canonical Gγs (DEP1 and GGC2), when linked with RGB1, stimulate grain size, whereas another Gγ, GS3, represses the effect of DEP1 and GGC2 to reduce the grain size by blocking the interaction of DEP1 and GGC2 with RGB1 (Sun et al., 2018). A similar decrease in grain weight is observed in Gα-null and Gβ-RNAi rice seeds when compared to their WT counterpart which suggests that both Gα and Gβ are positive regulators of grain size growth (Urano et al., 2020). Loss of rice Gα subunit (RGA1) confers dwarfness (Fujisawa et al., 1999), high salinity resistance (Yadav et al., 2012; Colaneri et al., 2014; Jangam et al., 2016). In our earlier study, Gβ phenotypes such as leaf length, seed area, grain length, grain weight, and number of crown roots are strongly correlated with RGA phenotypes. However, some other phenotypes of Gβ-RNAi rice such as embryo area, more severe root and cell death phenotypes, and leaf width cannot be explained by RGA functions (Urano et al., 2020). These results are consistent with Gβ function that forms a complex with either Gα or XLG, allowing a selective activation of downstream pathways. Because rice XLG3 (OsXLG3) is excluded from our analysis, here we consider rice XLG1, 2, and 4 (OsXLG1, OsXLG2, OsXLG4), keeping with the nomenclature established by Iwasaki et al. (2008). It should be noted that Cui et al. (2020) deviated from this nomenclature by designating these extra-large G proteins as PXLG1-4 with no strict correspondence in gene number to the established nomenclature.

Based on the phenotypical characterizations of rice Gα-null and Gβ-RNAi mutants (Urano et al., 2020; Wu et al., 2020), we hypothesized that some agronomical traits observed in Gβ-RNAi line but not in Gα-null mutant are associated with XLGs. In *Arabidopsis*, XLGs (AtXLGs) complement the function of Gα (Urano et al., 2016) and regulate root morphology, stress responsiveness, and cytokinin-induced development (Urano et al., 2016; Liang et al., 2017), whereas the canonical Gα plays a wide range of developmental roles (Urano et al., 2016). Unlike Gα, the cellular localization of XLGs is partner-dependent (Liang et al., 2017). AtXLG2 interacts directly or indirectly with plasma membrane localized receptor-like kinase [RLK, FLAGELLIN-SENSING 2 (FLS2) and receptor-like cytoplasmic kinase BIK1 (Liang et al., 2016)]. Cui et al. (2020) used CRISPR/Cas9 to generate single *Osxlg* mutants and found various phenotypic changes in yield components and stress tolerance, especially under salinity stress. The mutant of *pxlg4* (*Osxlg4*) showed improved stress tolerance with drought, chilling, and salinity compared to wild type (WT) and other mutants of *OsXLGs*. Simultaneously, all four mutants exhibited an early heading phenotype. The *plxg1* mutant had a shorter panicle. However, the number of panicles increased compared to the controls resulting in an increase in grain yield per *plxg1* mutant than WT. Unfortunately, it is not clear whether the alleles are null mutations (Cui et al., 2020). In maize, however, mutations of the three *XLG* genes were shown to be complete loss-of-function alleles and their combination confers seedling lethality, enhanced cell death-presumably programmed, and unregulated expression of immunity-related genes (Wu et al., 2018). This recapitulates the phenotypes of the rice and maize Gβ null mutants (Utsunomiya et al., 2011; Sun et al., 2018; Urano et al., 2020; Wu et al., 2020).

We genetically ablated all three rice *XLGs* individually and in combination taking advantage of the fact that CRISPR/Cas9-mediated genome editing enables researchers to examine the function of multiple genes at once by simultaneously targeting and mutating multiple genomic loci in a single experiment (Cong et al., 2013; Xie et al., 2015). A total of 26 new alleles are made accessible for the rice community. A comprehensive characterization of agronomical traits revealed that individual loss of rice *XLG* genes confer increased grain size, especially grain from *Osxlg2* and *Osxlg4* lines, and a better plant growth phenotype but it has a detrimental effect on grain filling. Simultaneously, combination of *Osxlg1*&*4* with loss-of-function alleles in double mutant exhibited salinity tolerance. Moreover, loss of all three *OsXLG* genes decreases fitness probably through conferring hypersensitivity to pathogens.

## Materials and Methods

### Plant Materials and Growth Conditions in the Greenhouse and Axenically

Rice (*Oryza sativa*) seeds of wild-type Nipponbare (WT-NIP) and CRISPR/Cas9-induced *Osxlg* null mutants were germinated on petri dishes layered with moist filter papers by incubating at 30°C in the dark for 3 days. Germinated seeds of WT and *Osxlg* mutants were transferred to soil and grown in a greenhouse under a 10-h light or 14-h dark cycle at 28°C (day) and 22°C (night). Plants were grown in greenhouses at the University of North Carolina at Chapel Hill, University of Georgia in Athens, GA and Temasek Life Sciences Laboratory in Singapore. Rice seedlings were planted in 0.5 L (0.13 gallons) pots for 5 weeks. Rice plants were grown in a mixture of two parts Fafard 3B, one-part Concrete Sand, and Osmocote plus granular fertilizer. Fertilizer applied at planting was Sprint 330 Iron Chelate and Jack’s Peat Lite Special 20-10-20 (nitrogen–phosphorus–potassium) as per Biswal et al. (2018). The *Osxlg* single, double, and triple mutants were grown in three different rows with their respective WT Nipponbare control plants for the growth analysis. The landrace TP309 was used as an additional control to ensure that the WT plants were growing well in the above greenhouse conditions as we have seen earlier (Biswal et al., 2018).

For the measurement of growth parameters, 5-week-old plants from both control and *Osxlg* mutants were propagated in 3.8 L (1 gallon) pots for 90 days. High salinity resistance was tested axenically. Rice seeds were sterilized and germinated for 7 days on 1/2 × Murashige-Skoog (MS) plates with 1% agar and subsequently transferred into Falcon tubes containing 50 mL 1/4 × MS solutions with or without 125 mM NaCl for 8 days. Growth chamber condition was a 16-h light or 8-h dark cycle at 28°C (day) and 26°C (night) with 70% humidity. Solutions were changed every 2 days to avoid any precipitation and contamination. Shoot and root lengths were quantitated, and rice images were taken at day 6 and 8 days of treatment.

### Generation of Transgenic Lines

The binary vector pRGEB32 (Plasmid # 63142) and cloning vector pGTR (Plasmid # 63143) were obtained from AddGene (Xie et al., 2015). The unique 20-nucleotide spacer sequences (*OsXLG1*: GGCTGCGCGGTGGGAATCGC, *OsXLG2*: CGGTGGGCCGTCGTACTCTA, and *OsXLG4*: CCGGCCGCCCTATGCAGTCC) were identified by submitting the MSU Id of *XLG* genes to the CRISPR-PLANT server (Xie et al., 2014). Oligos were synthesized and cloned into pRGEB32 binary plasmid as described by Xie et al. (2015). The polycistronic tRNA–gRNA (PTG) parts were amplified using one or more pair of these primers while using plasmid pGTR as template. Finally, the PTGs were introduced into the binary vector pRGEB32 by Golden Gate assembly using type IIS restriction enzyme *Bsa*I. We also used Cas-OFFinder^1^ (Bae et al., 2014), to select guide sequences that have almost no off-targets. We made all combinations of constructs for all three *XLG* genes (*XLG1, XLG2, and XLG4*). *Agrobacterium*-mediated transformation was used to transform calli derived from Japonica rice “Nipponbare” seeds. Control Nipponbare plants had been through the same tissue culture and regeneration process but did not contain the transgene. Transgene integration was verified by PCR analysis using primer pairs OsUbi_1051F/Cas9_868R (Supplementary Table 2).

### Mutation Analysis

Genomic DNA was extracted from 2 to 3-cm-long young leaf using a modified CTAB protocol (Rajendrakumar et al., 2006). Target sequences were amplified by Phusion DNA polymerase^®^ (New England Biolabls, United States) using gene-specific primers flanking the target site (Supplementary Table 2) and standard procedure. The amplicons were initially analyzed by Surveyor mutation detection assay^®^ (IDT, United States) as per supplier’s instruction to detect mutants and their zygosity. DNA amplicons from homozygous mutants were Sanger sequenced to confirm the nature of mutations.

#### Phenotyping and Measurement of Seed Weight

Three-day-old seedlings of WT and *Osxlg* mutants were transferred to soil in the greenhouse. The *Osxlg* single, double, and triple mutants were grown in three different rows with their respective WT plants. Each row was 2 feet apart from each other for optimum growth and development. Six to 20 plants from WT and *Osxlg* mutants were measured for plant height and tiller number. For dry weight measurements, the entire above ground shoot parts of 85–100-day-old WT and *Osxlg* mutants were harvested and dried at 70°C for 5 days and weighed (*N* = 5–13) (Biswal et al., 2018). All plants were grown to maturity. Harvested seeds were air-dried and stored at room temperature. The weight of fully filled dried seeds was quantitated using 50–200 seeds.

### Disease Assays

For disease assays with *Magnaporthe oryzae* (*M. oryzae*), rice plants were grown in the greenhouse, in plastic pots (9 × 9 × 9, 5 cm) filled with substrate (58% blond peat, 18% coconut powder, 10% perlite, 9% volcanic sand, and 5% clay) supplemented with 3,5 g/L of fertilization (Basacote Native 6 M, NPK 14-3-19) under 16-h artificial light (55,000 lumen) at 27–23°C as described earlier (Pélissier et al., 2021). We chose the GY11 strain of *M. oryzae* as it shows moderate virulence on Nipponbare (Delteil et al., 2012). GY11 was grown for 10 days on rice flour agar medium (20 g of rice flour, 15 g of agar, 2.5 g of yeast extract, and 1 L of distilled water) under fluorescent light (12 h a day) at 26°C. We harvested conidia by flooding the plate with 5 ml of sterile distilled water. Thirty milliliters from a suspension of 1,00,000 conidia per mL (with 0.1% gelatin) were sprayed on each tray containing 15 pots (∼ 25,000 conidia or plant) on 3-week-old plants as described in Berruyer et al. (2003).

After inoculation, rice plants were incubated for 16 h in a controlled climatic chamber at 25°C with 95% relative humidity and later returned to normal growth conditions. Six days after inoculation and before epidemics could start, we scanned at the same resolution (600 pixels per inch) the symptoms of the last, well-developed leaf of 3–4 focal plants per pot. Abnormal plants were not scored and pots with less than three focal and three neighbor were not counted. The pictures were analyzed by LeAFtool (Lesion Area Finding tool), an R package developed in-house and available on github depository.^2^ The program measures lesion number and leaf area. Parameters used for picture analysis were at least 10, 000 pixels for leaves and 50 pixels for lesion areas, with a blur at 1. To account for outliers and software mistakes, we removed lesions with aberrant size from the analysis. Finally, leaf susceptibility was estimated by the number of a lesion per cm^2^ of leaf area.

### Statistical Analysis

Statistical analysis was performed using Statistica 5.0. The significance of differences between control (WT) and *Osxlg* mutants was analyzed using a one-way ANOVA followed by Tukey’s multiple comparison or *post hoc* test.

## Results

### Phylogeny of *Oryza sativa* Extra-Large G Proteins

**FIGURE 1:**
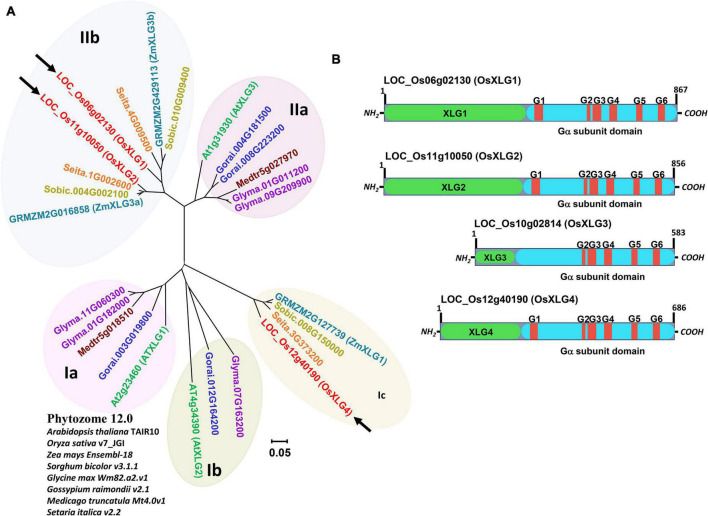
Phylogenetic tree and protein structure of rice XLGs. **(A)** Phylogeny of XLG protein sequences. Sequences were retrieved from *Arabidopsis thaliana* TAIR10 (green), *O. sativa* v7.0 (red), *Zea mays Ensembl-18* (turquoise), *Sorghum bicolor v3.1.1* (dark yellow), *Glycine max Wm82.a2.v1* (purple), *Gossypium raimondii v2.1* (blue), *Medicago truncatula Mt4.0v1* (dark red), and *Setaria italica* (orange) from Phytozome 12.0 (https://phytozome.jgi.doe.gov/). LOC_Os06g02130, LOC_Os11g10050, and LOC_Os12g40190 (in red fonts with black arrow) are named in this paper as OsXLG1, OsXLG2, and OsXLG4. The tree was constructed by the neighbor-joining method using MEGA7 (Kumar et al., 2016). **(B)** Schematic diagram of rice XLG domain architecture. Domains unique to the XLG family are shown in green boxes. Turquoise color represents homologous Gα subunit sequence. Conserved amino acid sequences shown in red rectangle with G#s represent motifs involved in GTP-binding.

Phylogenetic analysis of XLGs from *Arabidopsis thaliana*, rice, and six other crops (maize, sorghum, soybean, cotton, alfalfa, and foxtail millet) revealed two major clades based on the full-length protein sequence, with the clade **I** and **II** further divided into three and two subclades, respectively (Figure 1A). Rice XLG1 and XLG2 (OsXLG1 and OsXLG2) belong to clade **IIb** whereas XLG4 (OsXLG4) belongs to clade **Ib** (Figure 1A). Besides the presence of one canonical Gα (not shown), four OsXLGs (XLG1-XLG4) have been reported earlier in rice genome (Iwasaki et al., 2008; Figure 1B). Rice XLG3 (OsXLG3) was excluded from the phylogenetic analysis due to the low sequence similarity to other entries (see “Discussion” section).

### Generation of *Oryza sativa* Extra-Large G Protein Mutants

Approximately 30 independent transgenic events each were generated for *Osxlg1*, *Osxlg2*, *Osxlg4, Osxlg1*&*2*, *Osxlg1*&*4*, *Osxlg2*&*4*, and *Osxlg1*,*2*&*4* gene combinations by transforming embryogenic rice calli with the corresponding constructs in the background of Nipponbare (NIP). All transgenic lines showed mutations in the target genes in the T0 generation. Homozygous mutants were identified in the T0 generations for *OsXLG1* gene and in the T1 generation for the remaining events. However, we did not observe any plant with homozygous mutations in both *OsXLG1* and *OsXLG2* genes together. The *Osxlg4* mutants were selected from a transgenic line generated by the *Osxlg1*&*4* CRISPR cassette that did not show the presence of mutation in *Osxlg1* and were made Cas9 free.

**FIGURE 2:**
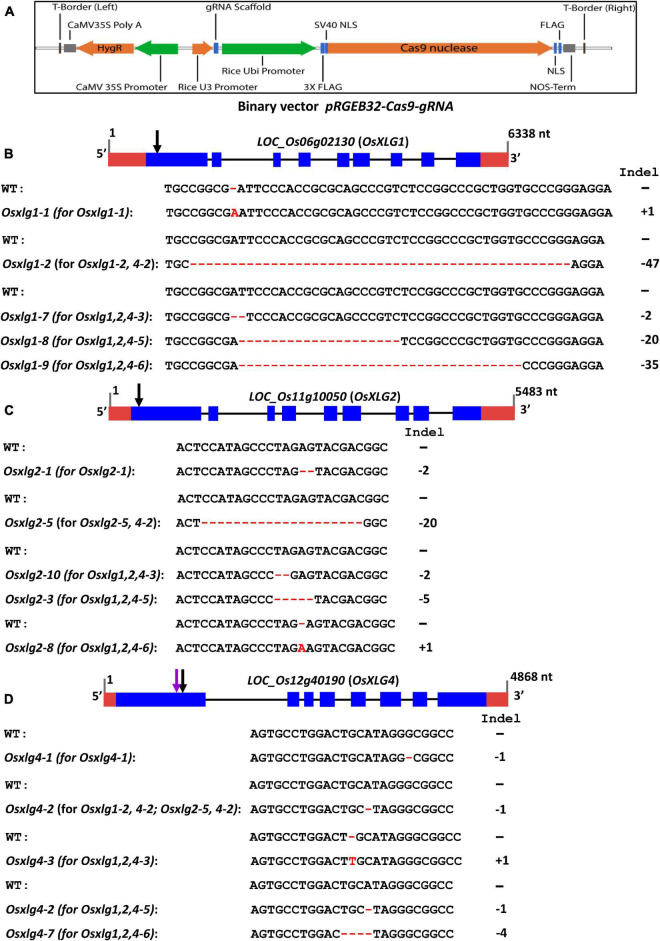
Rice *OsXLG*s gene model, CRISPR/Cas9 vector map, positions of the guide RNAs (gRNA) and mutations. **(A)** A schematic of the binary vector along with *Cas9* and position of gRNA (pRGEB32-Cas9-gRNA). **(B–D)** Rice *OsXLG1* (*LOC_Os06g02130*), *OsXLG2* (*LOC_Os11g10050*), and *OsXLG4* (*LOC_Os12g40190*) gene model from Phytozome 12.0 *Oryza sativa v7_JGI*. nt, nucleotides. Blue boxes indicate exons, black lines indicate introns, and red boxes are the 5′ and 3′ untranslated regions (UTRs). The indel patterns are shown in red color. Black arrows indicate the position of the nucleotide deletion and substitution mutations in the *OsXLG1*, *OsXLG2, OsXLG4* single, double, and triple mutant, whereas purple arrow indicates mutation of *OsXLG4* in double mutants **(A–C)**.

Most of the mutations generated were indels that created frameshifts that led to a premature stop codon located in the N-terminal domain of XLG (Supplementary Table 3, Table 1, and Figure 2) such that each of these alleles is predicted to be loss-of-function alleles. We selected three independent single mutants (*Osxlg1-1, Osxlg2-1, and Osxlg4-1*), two double mutants (*Osxlg1-2, 4-2*; *Osxlg2-5, 4-2*), and three triple mutants (*Osxlg1,2,4-3*; *Osxlg1,2,4-5*; *Osxlg1,2,4-6*) from the T1 generation for further agronomical traits analysis. The alleles associated with three single mutants are *Osxlg1-1* (1-bp insertion for *OsXLG1*), *Osxlg 2-1* (2-bp deletion for *OsXLG2*), *and Osxlg4-1* (1-bp deletion for *OsXLG4*) whereas *Osxlg1-2* (47-bp deletion for *OsXLG1*), *Osxlg2-5* (20-bp deletion for *OsXLG2*), *and Osxlg4-2* (1-bp deletion for *OsXLG4*) alleles are linked with two double mutants (Table 1 and Figures 2B–D). We identified null alleles (*Osxlg1-7*, 2-bp deletion, *Osxlg1-8*, 20-bp deletion, *Osxlg1-9*, 35-bp deletion for *OsXLG1; Osxlg2-3*, 5-bp deletion, *Osxlg2-8*, 1-bp insertion, *Osxlg2-10*, 2-bp deletion for *OsXLG2*; *Osxlg4-2*, 1-bp deletion, *Osxlg4-3*, 1-bp insertion, *Osxlg4-7*, 4-bp deletion for *OsXLG4*) of all three genes within the N-terminal half of the protein-coding region in three triple mutants (Table 1 and Figures 2B–D).

**TABLE 1:** Novel *Osxlg* alleles.

Allele	Indel or substitution	Non-sense or missense mutation
*Osxlg1*-1	One base insertion.	Frameshift mutation leading to STOP codon at residue 111.
*Osxlg1*-2	Deletion of 47 bases.	Frameshift mutation after residue 58 leading to a stop codon at residue 111.
*Osxlg1*-7	Deletion of two bases.	Frameshift mutation at residue 61 leading to a stop codon at residue 110.
*Osxlg1*-8	Deletion of 20 bases.	Frameshift mutation at residue 61 leading to a stop codon at residue 104.
*Osxlg1*-9	Deletion of 35 bases.	Frameshift mutation at residue 61 leading to a stop codon at residue 99.
*Osxlg2*-1	Deletion of two bases.	Frameshift mutation leading to a STOP codon at residue 47.
*Osxlg2*-3	Deletion of five bases in the target site.	Frameshift mutation at residue 37 followed by a STOP codon at 45.
*Osxlg2*-5	Deletion of 20 bases at the target site.	Frameshift mutation leading to two STOP codons near residue 46.
*Osxlg2*-8	One base insertion at the target site.	Frameshift mutation from residue 38 and a STOP codon at residue 47.
*Osxlg2*-10	Deletion of two bases.	Frameshift mutation from residue 37 leading to a STOP codon at residue 46.
*Osxlg4*-1	Deletion of one base at the target site.	Frameshift mutation at residue 272 leading to a STOP codon at residue 303.
*Osxlg4*-2	Deletion of one base at the target site.	Frameshift mutation after residue 270 leading to a stop codon at residue 271.
*Osxlg4*-3	One base insertion.	Frameshift mutation from residue 270 leading to a STOP codon at residue 343.
*Osxlg4*-7	Deletion of four bases.	Frameshift mutation from 270 generated an immediate STOP codon.

### Grain Size and Seed Filling in Rice *Oryza sativa* Extra-Large G Protein Mutants

**FIGURE 3:**
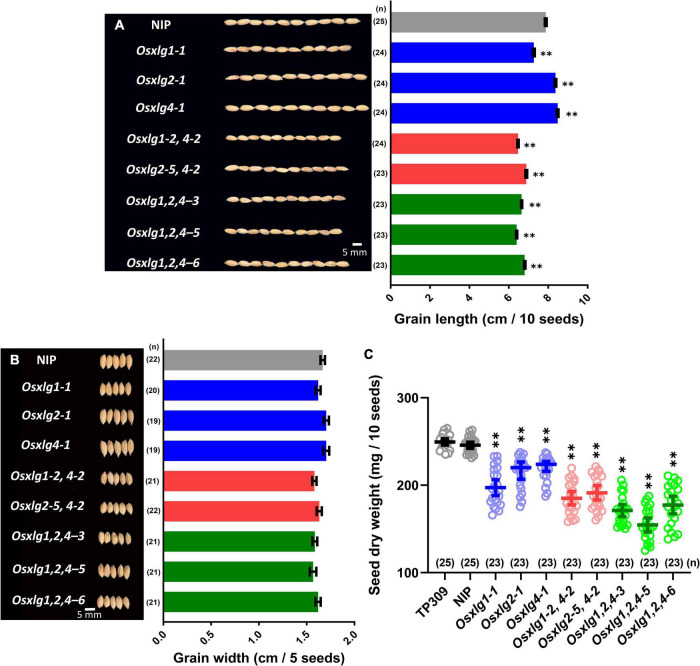
Rice seed phenotypes and dry weight in *Osxlg* mutants. Grain and grain length **(A)**, grain width **(B)**, and seed dry weight **(C)**, of WT cv. Nipponbare (NIP) and Taipei (TP309) and *Osxlg* single, double, and triple mutant plants. Each data point represents raw value of 10 seeds’ weight, the horizontal lines indicate the means, and the error bars represent 95% confidence intervals. *Oryza sativa* L. japonica cv. Taipei 309 (TP309) was used as an additional control. The sample size was shown as (*n*). Statistical analysis was done using one-way ANOVA followed by Tukey’s multiple comparison test, ^**^p < 0.001 by comparing WTNIP with *Osxlg* mutants. Bar = 5 mm.

To compare the effects of these alleles on grain size, we checked the grain size in the mature panicles. Two single mutants had longer grain length (*Osxlg2-1*, 6%; *Osxlg4-1*, 8%) compared to WT (Figure 3A). The *Osxlg1-1* had shorter grain length compared to WT (Figure 3A). This contrasts with the report on *pxlg1* mutant lines which had increased grain length (Cui et al., 2020). There was a significant decrease in grain length in the double (13–18%) and triple (14–19%) mutants compared to WT. These results indicate that XLG2 and XLG4 each individually negatively repress grain size while the combination of XLG1, XLG2, and XLG4 proteins reduces the grain size in rice. This indicates a complicated epistasis as will be proposed in “Discussion” section. Aside from the change in grain size, no other differences were observed in grain width and phenotype in the *Osxlg* alleles compared to the WT (Figure 3B). These results suggest that XLGs selectively modulate grain length in rice.

### *Oryza sativa* Extra-Large G Proteins Are Essential for Seed Filling in Rice

The seed or grain weight is another important agronomic trait in rice. The single *Osxlg* mutants showed 11–20% significantly reduced (*Osxlg1-1*, 20%; *Osxlg2-1*, 13%; and *Osxlg4-1*, 11%; Figure 3C) seed dry weight compared to WT. The seed weight in both double (*Osxlg1-2,4-2*, 25%; *Osxlg2-5,4-2*, 22%) and triple mutants (*Osxlg1,2,4-3*, 30%; *Osxlg1,2,4-5*, 37%; *and Osxlg1,2,4-6*, 28%) were also significantly lower than WT (Figure 3C). The Gα-null and Gβ-RNAi rice seeds displayed similar weight loss compared to WT (Urano et al., 2020). The reduced seed dry weight in all the *Osxlg* alleles indicates that XLGs are essential for grain weight in rice.

### Extra-Large G Proteins Are Associated With Panicle Phenotype and Grain Number

**FIGURE 4:**
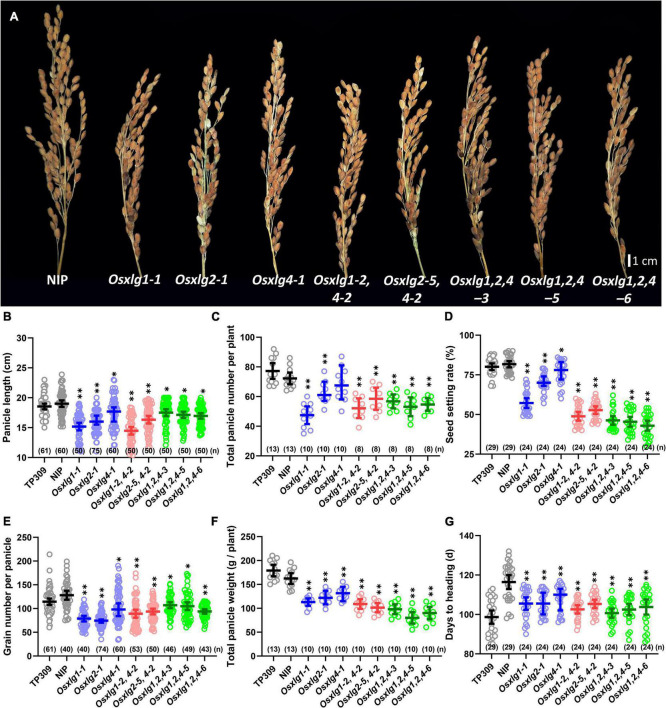
Panicle phenotype of rice *Osxlg* plants. Genetic effect of *Osxlg* mutations on panicle size and grain yield. Panicle images **(A)**, panicles length **(B)**, total number of panicle per plant **(C)**, seed setting rates **(D)**, grain number per panicle **(E)**, total panicle weight per plant **(F)**, and days to heading **(G)** from controls and *Osxlg* single, double, and triple mutants. Significant differences between WT-NIP and *Osxlg* mutants were determined with one-way ANOVA with the *p*-values that are expressed as *p < 0.05, ^**^p < 0.001. (*n*) = sample size, Bar = 1 cm.

Panicle length is one characteristic of panicle architecture and is measured as a yield-related trait in rice. The panicle length of *Osxlg* mutants was significantly reduced (single mutants, 9–20%; double mutants, 14–24%; and triple mutants, 8–11%) compared to WT (Figures 4A,B). Similarly, all the *Osxlg* mutant lines significantly reduced the number of total panicles per plant except *Osxlg4-1* (Figure 4C). The panicle length and seed setting rate were combined to determine the number of grains per panicle. All the *Osxlg* mutant lines analyzed showed a significant decrease (6–47%) in seed setting rate compared to WT (Figure 4D). Simultaneously, there was a reduction in the grain number per panicle in all the *Osxlg* mutant lines (single mutants, 18–41%; double mutants, 27–30%; and triple mutants, 16–27%), which led to a decrease in the total panicle weight per plant (19–51%) compared to that the WT (Figures 4E,F). We also observed that all the *Osxlg* alleles reached “Days to Heading” significantly (7–14%) earlier than the WT (Figure 4G). The panicle phenotype and reduced grain number observed in this study indicated that the XLG signaling participates in the control of plant heading, panicle size, number, and grain number per panicle in rice.

### The *Oryza sativa* Extra-Large G Protein Mutation Modulates Plant Growth in Rice

**FIGURE 5:**
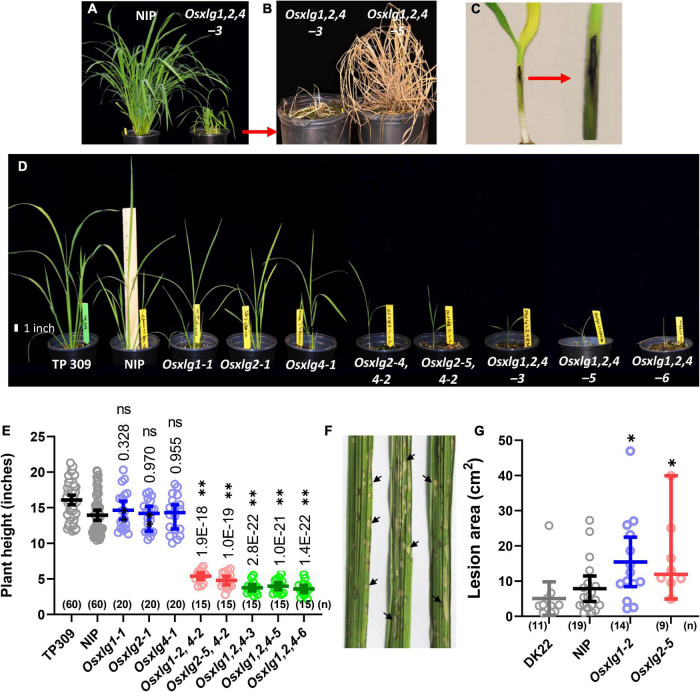
Pathogen detection and growth phenotype of *Osxlg* mutant at seedling stage. Eighty-five-day-old NIP and triple mutant (*Osxlg1,2,4-3*) plants **(A)**, the dead triple mutant plants (*Osxlg1,2,4-3, Osxlg1,2,4-5*) **(B)**, pathogen infection at the seedling stage **(C)**, growth phenotypes of 5-week-old rice WT-NIP, *Osxlg* single, double, and triple mutant plants in 0.5-L pots **(D)**, the plant height of *Osxlg* mutants **(E)** lines in comparison with the controls (WT-NIP). Six pots of each genotype (NIP, *Osxlg1-2*, *Osxlg2-5*) were used to measure lesion density caused by *M. oryzae* inoculation (isolate GY11). GY11 isolates provoke grayish lesions **(F)** at the center of which the fungus sporulates. Black arrows indicate grayish lesions. Lesion densities **(G)** were significantly higher in *Osxlg* mutants than in WT-NIP. DK22 genotype used as internal control. Asterisks indicate values significantly different from WT-NIP by ANOVA followed by Tukey’s multiple comparison test, *p < 0.05, ^**^p < 0.001, (*n*) = sample size. ns, not significant.

**FIGURE 6:**
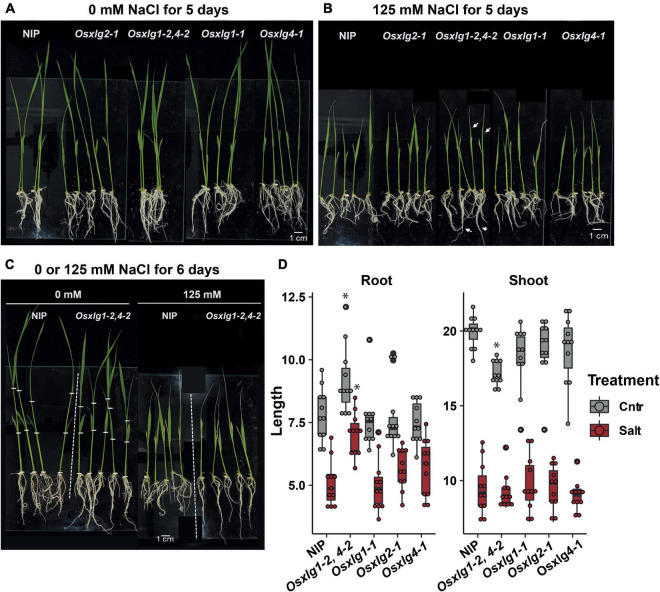
Root and shoot length of *Osxlg* mutants under salt stress condition. Seven-day-old hydroponically-grown rice seedlings were treated with 0 mM (Control, gray box) **(A)**, or 125 mM (Salt, red box) **(B)**, NaCl for 6 days **(C)** with root and shoot length **(D)**. Sample size = 10. Box plots show 1st and 3rd quantile. Significant differences were determined with two-way ANOVA followed by Tukey’s *post hoc* test. Different lowercase letters (a–d) indicate significant differences across genotypes.

Combining null mutations in all three *OsXLG* genes greatly decreased fitness. Plants grown in the greenhouse tillered to different degrees then died (Figures 5A,B). The presence of disease on these plants (Figure 5C) suggested that these triple mutants are hypersensitive to pathogens and could be related to autoimmunity as previously proposed (Urano et al., 2020). As shown in Figure 6A, triple mutants grown axenically were the same height as its landrace, Nipponbare (NIP). The survival rate of triple mutants in the first 3 weeks on the soil was less than 3%. The severity of the diseased phenotype (lethality) of the triple *Osxlg* mutants prompted a quantitative analysis of disease susceptibility of the single *Osxlg* mutants. Lesions on leaves (arrows Figure 5F) were counted and the number expressed per area. When treated with rice blast spores as described in section “Materials and Methods,” *Osxlg1-2* and *Osxlg2-5* had significantly more lesions than the WT (Figure 5G).

Null mutations in individual *OsXLG* genes did not confer a reduction in plant height at the juvenile stage (35 days) compared to its landrace NIP consistent with the alleles described by Cui et al. (2020) (Figures 5D,E). In contrast, substantial morphological differences were observed between the WT (NIP) and combinatorial *Osxlg* mutants (Figure 5). The plant height of 35-day-old *Osxlg* double and triple mutant lines was significantly lower by 61–66% and 71–74% (Figures 5D,E), respectively, compared to WT. These results suggest that XLG loss-of-function mutants play a significant role in postgermination growth at the early stage of vegetative development in rice. We do not rule out an indirect role in growth such as autoimmunity as previously proposed (Urano et al., 2020).

Interestingly, 100-day-old *Osxlg* single mutants showed 7–12% (average 10%, *p* < 0.05–0.01) increase in plant height compared to WT plants (Supplementary Figures 1A, D). In addition, these mutants had 15–21% (average 18%, *p* < 0.01) increased tiller number compared to WT (Supplementary Figure 1G). In contrast, reduced plant height was observed in double (*Osxlg1-2, 4-2*, 29%; *Osxlg2-5, 4-2*, 26%; average 28%, *p* < 0.01; Supplementary Figures 1B,E) and triple mutant (*Osxlg1,2,4-3, 41%*; *Osxlg1,2,4-5, 35%*; *Osxlg1,2,4-6*, 38%, average 38%, *p* < 0.01; Supplementary Figures 1C,F) lines including significant reduction in tiller numbers in double (average 36%, *p* < 0.01, Supplementary Figure 1H) and triple mutant (average 42%, *p* < 0.01, Supplementary Figure 1I) lines compared to WT. To establish the growth kinetics of *Osxlg* mutants, we performed a time-course analysis of *Osxlg* mutants from weeks 4 to 12 (Supplementary Figures 2, 3). Measurement of plant height and tiller numbers over a 12-week growth period showed that the increase in height and tiller numbers of *Osxlg* single mutants were statistically supported starting at weeks 8 and 6, respectively (Supplementary Figures 2A, 3A). Difference in growth pattern (reduced plant heights and number of tillers) could be observed as early as week 4 in double (Supplementary Figures 2B, 3B) and triple mutant (Supplementary Figures 2C, 3C) lines, which continued.

### The *Oryza sativa* Extra-Large G Protein Single Mutation Increases Biomass Yield in Rice

**FIGURE 7:**
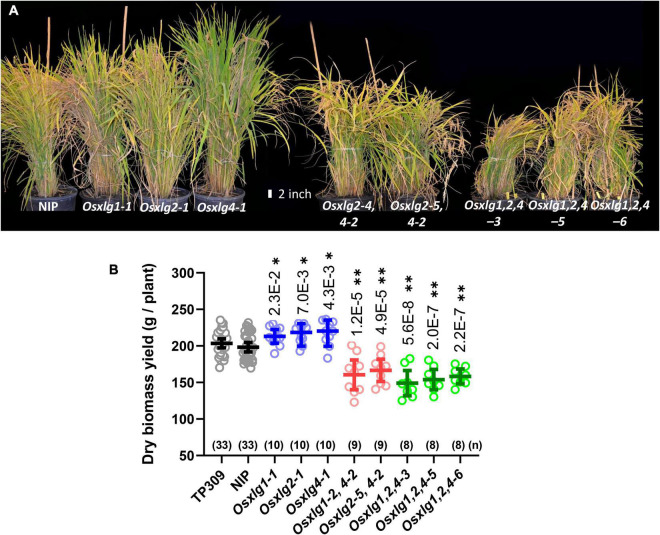
Growth phenotypes and dry aerial weight of rice *Osxlg* plants. Growth phenotypes and dry aerial biomass weight of 125-day-old rice WT NIP, *Osxlg* single, double, and triple mutant greenhouse grown plants **(A)**. The raw values of controls and *Osxlg* mutants are shown in **(B)**. The horizontal lines indicate the means, the error bars represent 95% confidence intervals, and (*n*) is the sample size. Significant differences between WT-NIP and *Osxlg* mutants were determined by statistical analysis using Statistica 5.0 with one-way ANOVA followed by Tukey’s multiple comparison test. *p-*values are expressed as *P < 0.05, ^**^P < 0.001.

All *Osxlg* single mutants showed increased growth compared to WT in the greenhouse. The increased plant height (Supplementary Figures 1A,D, 2A) and significantly more tillers (Supplementary Figures 1G, 3A) beginning in week 4 resulted in 7–10% (average 9%, *p* < 0.01) more aerial biomass in mature *Osxlg* single mutant lines than WT (Figures 7A,B). Inhibition of vegetative growth was observed in both double and triple *Osxlg* mutants starting from early seedling growth stage (Figures 5D,E) compared to the controls. This growth inhibition led to an overall 16–19% (average 18%, *p* < 0.01) and 20–25% (average 23%, *p* < 0.01) decrease in total aerial dry biomass of the greenhouse-grown *Osxlg* double and triple mutants (Figures 7A,B), respectively. Rice and maize differ in that loss of any single maize *XLG* gene function has no effect on plant height or development although loss of two of the three confers a scorable reduction in growth (Wu et al., 2018).

### Comparison of Agronomic Traits of *Oryza sativa* Extra-Large G Protein Mutants Obtained at a Tropical Climate

**FIGURE 8:**
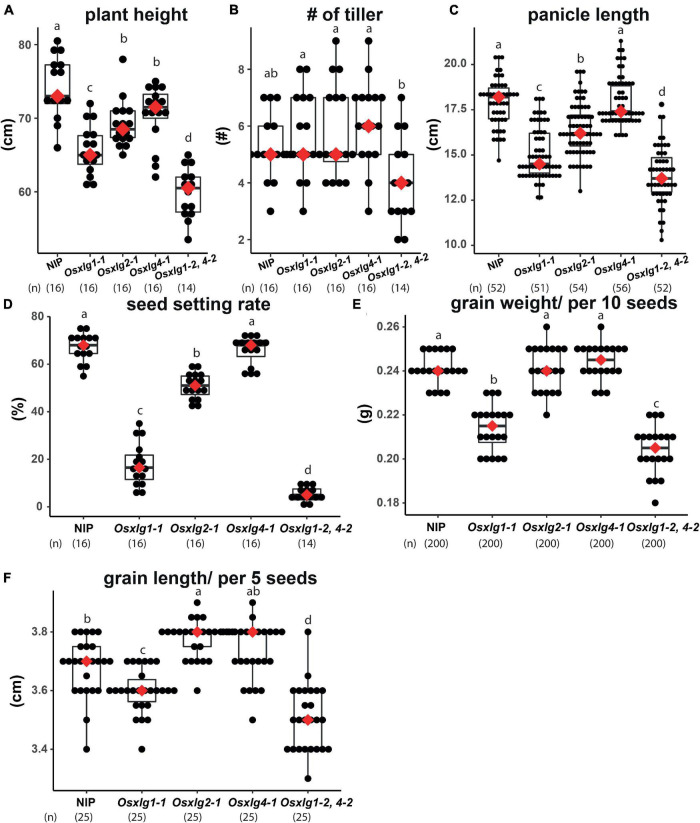
Agronomic traits of *Osxlg* mutants under tropical greenhouse condition. Basic agronomic traits of *Osxlg* single (*Osxlg1-1, Osxlg2-1, Osxlg4-1)*, double (*Osxlg1-2,4-2*) mutants, and WT (NIP) plants obtained in greenhouse condition. Plant height **(A)**, number of tillers **(B)**, panicle length **(C)**, seed setting rates **(D)**, grain weight per 10 seeds **(E)**, and grain length per five seeds **(F)** from controls and *Osxlg* single and double. Box plots showed 1st and 3rd quantile. Red dots represent median. Sample size was shown as (*n*). Significance values are with one-way ANOVA followed by Tukey’s *post hoc* test.

Agronomical traits of *Osxlg* mutants were further confirmed under tropical greenhouse conditions (Singapore) where day and night temperatures fluctuate roughly at 32 and 25°C as the average temperature. All the three *Osxlg* single mutants exhibited 5–12% shorter plant height (Figure 8A), although no differences were observed in a normal greenhouse condition (Figure 5). Similarly, *Osxlg1,4* double mutant showed more server defect of shoot growth (20% shorter height) in tropical greenhouse compared to the normal condition. We next measured tiller number, panicle length, seed setting rate, and seed weight and length (Figures 8B–F). Similar to our earlier observation, two out of three *Osxlg* single mutants (*Osxlg1* and *Osxlg2*) exhibited shorter panicle length by 12–17% as compared to NIP landrace (Figure 8B). Poor seed setting rate in single *Osxlg1* and double *Osxlg1,4* mutants appeared more severely under tropical condition (Figure 8D, 20 and 5%, respectively) compared to normal condition (Figure 4). The reduction in grain weight and length in *Osxlg1* single and *Osxlg1,4* double mutants were comparable to those under normal condition (Figures 8E,F). The deviation due to the normal and tropical greenhouse conditions suggests that some of developmental functions of XLGs are conditional rather than only controlling intrinsic developmental processes.

### The *Oryza sativa* Extra-Large G Protein Double Mutant Increases Salinity Tolerance in Rice

The conditional developmental phenotypes of rice *Osxlg* mutants were further tested under high salinity condition where both shoot and root growth were inhibited at young seedling stage. In a growth chamber maintaining temperature at 28°C, all the single *Osxlg* mutants showed comparable shoot and root lengths to NIP under non-salt and salt condition (Figure 6). However, the *Osxlg1,4* mutants exhibited significantly longer root lengths by 20 and 44% as compared to NIP plants under non-salt and salt stress condition, respectively (Figure 6D). In agreement with the data from greenhouse condition (Figure 8), *Osxlg1,4* exhibited a compact shoot architecture without salt treatment. The shoot size of the double mutant was similar to NIP under salt stress, suggesting that *Osxlg1,4* mutation generated stress signaling internally, causing shoot growth retardation.

## Discussion

We generated multiple genomic alleles for rice *XLG* genes. Although grain size was decreased in the single, double, and triple *Osxlg* mutants, we identified two single mutants, *Osxlg2-1* and *Osxlg4-1*, that conferred longer grains than WT. We also found a significant increase in plant height (7–12%), tiller number (15–21%), and aerial dry biomass (7–10%). In addition, *Osxlg1-2, 4-2* double mutation improved high salinity tolerance.

Rice XLG3 was not included in our analysis due to the partial truncation of its N-terminal domain and sequence dissimilarity suggesting ongoing purification of this gene. In addition, sequence analysis led to our positing that OsXLG3 is not monophyletic to the atypical XLG proteins. Cui et al. (2020) generated rice *Osxlg3* mutant (*pxlg3*). However, the *pxlg3* plants exhibited no differences in panicle height, grain length, and 1,000 grain weight, suggesting that OsXLG3 possesses no or very limited functions in rice. The *pxlg3* phenotypes contrast to our experimental evidence that single *Osxlg1*, *2*, and *4* mutations conferred significant effects on multiple traits such as reduced seed or grain weights and increased grain length (Figures 3A,C). Our statistically supported difference contrasts with Cui et al. (2020) who reported that *Osxlg1* mutants showed an increase in grain length and seed weight while other *Osxlg* mutations did not show any clear difference (Supplementary Table 4). We believe that this is due to differences in alleles whereas our alleles used for phenotyping are null and some of the alleles used by Cui and coworkers may not be. The phenotypic differences could be due to the use of two different genetic backgrounds between this study and Cui et al. (2020).

We found that genetic ablation of *OsXLG* singly increased plant height, biomass yield, and seed size, whereas loss of two or more of the *OsXLG* genes reduced these traits. Similarly, high salinity tolerance was only observed in *Osxlg1-2, 2-4* double mutant, but not in any of single *Osxlg* alleles (Figure 6). These lines of evidence suggest a complicated epistatic relationship among the *Osxlg* null alleles. This also may be due to genetic redundancy of *XLGs* in the rice genome because it explained by the similar expression patterns of *OsXLG1*, *2*, and *4* genes, obtained from meta-transcriptome data (Xia et al., 2017),^3^ in different rice tissues (Supplementary Figure 4). In addition to rice *XLG* mutations, various types of mutations in *RGA*, *DEP1*, and *GS3* genes were identified from agronomical QTLs for grain size, panicle density, and dwarfness. XLG control of grain weight (Figure 3) and therefore the combination of Gα, XLG, and Gγ can be used as gene targets to manipulate genetically rice grain size, length, and weight. Most physiological alterations observed in rice Gα and XLG null mutants were consistent with those of Gβ-RNAi lines, as Gβ forms a complex with either Gα or XLG. Based on phenotypic profiles of rice Gα-null, Gβ-RNAi (Urano et al., 2020), and *Osxlg* mutant plants, we hypothesize that Gβ function in grain weight control is mediated by XLG rather than the canonical Gα.

Extra-large GTP-binding protein interacts with the Gβγ dimer presumably in competition with interaction of the canonical Gα subunit with the dimer. A scenario where four subunits (one canonical Gα and three atypical extra-large Gα subunits, XLGs) compete to form the heterotrimer is dynamic. Although much less is known about XLGs than canonical Gα subunits, a comparison was found between Arabidopsis and rice XLGs where the Arabidopsis *xlg123* triple mutants (Urano et al., 2016) had leaf and seed phenotype that are similar to the *Osxlg* double and triple mutants (Figures 3, 5). The Arabidopsis quadruple (*gpa1 xlg123*) mutants showed the same seed phenotype, but the leaf growth was significantly reduced compared to the *xlg123* triple mutants (Urano et al., 2016). Although no rice quadruple mutant, null alleles of the genes encoding Gα and all three XLGs (*rga1 xlg124*), has been generated for this study, we also hope that they cannot survive because the *Osxlg* triple mutants are sick or may exhibit severe dwarf phenotype such as the Arabidopsis quadruple mutants. In addition, knocking out three *XLGs* in the *gpa1* background reduces the leaf and seed length (Urano et al., 2016), suggesting that their function with the canonical Gα is essential for controlling leaf and seed size. This also suggests that XLGs and Gα work independently for regulating various agronomical traits. We did not find salinity tolerance in *Osxlg2* or *Osxlg4* single mutants, instead the null combination of *Osxlg2* and *Osxlg4* genes showed resistance to salinity (Figure 6). In the presence of sodium chloride, Arabidopsis the *xlg123* triple null mutant had reduced leaf growth (Urano et al., 2016). Based on this report and an earlier study, our results provide solid evidence that XLG2 independently and in combination with XLG4 promotes salt resistance in rice (Urano et al., 2016; Cui et al., 2020).

The *Osxlg2-1* and *Osxlg4-1* single alleles exhibited a slight increase in grain length, while the *Osxlg1, 4* double mutants conferred resistance to high salinity. These traits conferred by a short indel mutation at *XLG* loci can potentially be inserted into agronomically useful germplasms. However, the increased sensitivity to disease observed in all three *Osxlg* mutants indicates that a sophisticated approach will be required for engineering increased yield through *XLG* silencing. Additional analysis may show that the increased yield can be obtained through reproductive tissue-specific silencing. Alternatively, the increased yield and hypersensitivity traits may be inextricably linked. For example, loss of the canonical Gα subunit in rice confers dwarfness (Fujisawa et al., 1999) which on its own can roughly double the harvest index through reduced lodging. However, the reduction in grain size and the hypersensitivity to rice blast disease also conferred by loss-of-function alleles of *RGA1* preempted the use of these alleles in agronomy. Nevertheless, these results indicate that systematic biotechnological manipulation of *XLG2* and *XLG4* genes can be used to develop bigger grain size to improve grain yield. Individual manipulation of *XLGs* is possible to achieve higher biomass and grain yield in rice, if the adverse effects for seed filling, seed dry weight, grain number per panicle, and panicle number per plant can be minimized.

## Data Availability Statement

The original contributions presented in the study are included in the article/Supplementary Material, further inquiries can be directed to the corresponding author/s.

## Author Contributions

AB, T-YW, DU, RP, J-BM, AMJ, and AKB designed, conceived, carried it out the experiment, and analyzed the data. AB, DU, J-BM, AMJ, and AKB wrote and edited the manuscript. All authors contributed to the article and approved the submitted version.

## Conflict of Interest

The authors declare that the research was conducted in the absence of any commercial or financial relationships that could be construed as a potential conflict of interest.

## Publisher’s Note

All claims expressed in this article are solely those of the authors and do not necessarily represent those of their affiliated organizations, or those of the publisher, the editors and the reviewers. Any product that may be evaluated in this article, or claim that may be made by its manufacturer, is not guaranteed or endorsed by the publisher.

## Footnotes

1
http://www.rgenome.net/cas-offinder/

2
https://github.com/sravel/LeAFtool

3
http://expression.ic4r.org/

## Supplementary Material

Supplementary Figure 1 Analysis of plant phenotype and development of *Osxlg* mutants at different time points. Growth phenotypes of 100-day-old rice WT NIP, *Osxlg* single **(A)**, 90-day-old WT-NIP and double **(B)**, and 70-day-old WT-NIP and triple mutant **(C)** plants. Ninety-five-day-old TP309 WT rice plant was used as an additional control in **(D–I)**. The raw values for height and number of tillers of greenhouse grown WT rice and *Osxlg* mutants are shown in **(D–I)**, respectively. The horizontal lines indicate the means, and the error bars represent 95% confidence intervals. *n* = sample size. Significance *p*-values are expressed as ^*^*p* < 0.05, ^**^*p* < 0.01. Significant differences between WT-NIP and *Osxlg* mutants were determined as calculated by statistical analysis using Statistica 5.0 with one-way ANOVA followed by Tukey’s multiple comparison test.

Supplementary Figure 2 Analysis of plant height of *Osxlg* mutants at different time points. Measurement of plant height **(A–C)** of *Osxlg-KO* mutants throughout the growth of the period from weeks 4 to 12 in comparison with the controls. Data are presented as box and whiskers plots showing the mean as “ + “ inside the boxes and the error bars represent 95% confidence intervals. For **(A)** *n* = 10 (TP309, NIP, *Osxlg1*), *n* = 12 (*Osxlg-2* and *Osxlg-4*); For **(B)** *n* = 10 (TP309, NIP), *n* = 12 (*Osxlg1-2, 4-2* and *Osxlg 2-5, 4-2*); For **(C)** *n* = 10 (TP309, NIP), *n* = 6 (*Osxlg1,2,4-3; Osxlg1,2,4-5; Osxlg1,2,4-6*). Significance *p-*values are expressed as ^*^*p* < 0.05, ^**^*p* < 0.01 by comparing WT-NIP with *Osxlg* mutants as calculated by statistical analysis using Statistica 5.0 with one-way ANOVA followed by Tukey’s multiple comparison test.

Supplementary Figure 3 Analysis of plant tiller growth and number of *Osxlg* mutants at different time points. Measurement of tiller numbers **(A–C)** of *Osxlg-KO* mutants throughout the growth of the period from weeks 4 to 12 in comparison with the controls. Data are presented as box and whiskers plots showing the mean as “ + “ inside the boxes and the error bars represent 95% confidence intervals. For **(A)** *n* = 10 (TP309, NIP, *Osxlg1*), *n* = 12 (*Osxlg-2* and *Osxlg-4*); For **(B)** *n* = 10 (TP309, NIP), *n* = 12 (*Osxlg1-2, 4-2* and *Osxlg 2-5, 4-2*); For **(C)** *n* = 10 (TP309, NIP), *n* = 6 (*Osxlg1,2,4-3; Osxlg1,2,4-5; Osxlg1,2,4-6*). *p*-Values are expressed as ^*^*p* < 0.05, ^**^*p* < 0.01. Significance differences between WT-NIP and *Osxlg* mutants were determined by statistical analysis using Statistica 5.0 with one-way ANOVA followed by Tukey’s multiple comparison test.

Supplementary Figure 4 Tissue-specific expression of *XLGs* in rice. Rice **(A)** *OsXLG1* (*LOC_Os06g02130*), **(B)** *OsXLG2* (*LOC_Os11g10050*), and **(C)** *OsXLG4* (*LOC_Os12g40190*) expression in different tissues. The expression data were extracted from the meta-transcriptome data available at http://expression.ic4r.org/ (Xia et al., 2017).
