# Supplementary figures and images for: Novel Mutant Alleles Reveal a Role of the Extra-Large G Protein in Rice Grain Filling, Panicle Architecture, Plant Growth, and Disease Resistance
Source: Front Plant Sci. 2022 Jan 3;12:782960. doi: 10.3389/fpls.2021.782960 (PMC8761985; doi:10.3389/fpls.2021.782960)

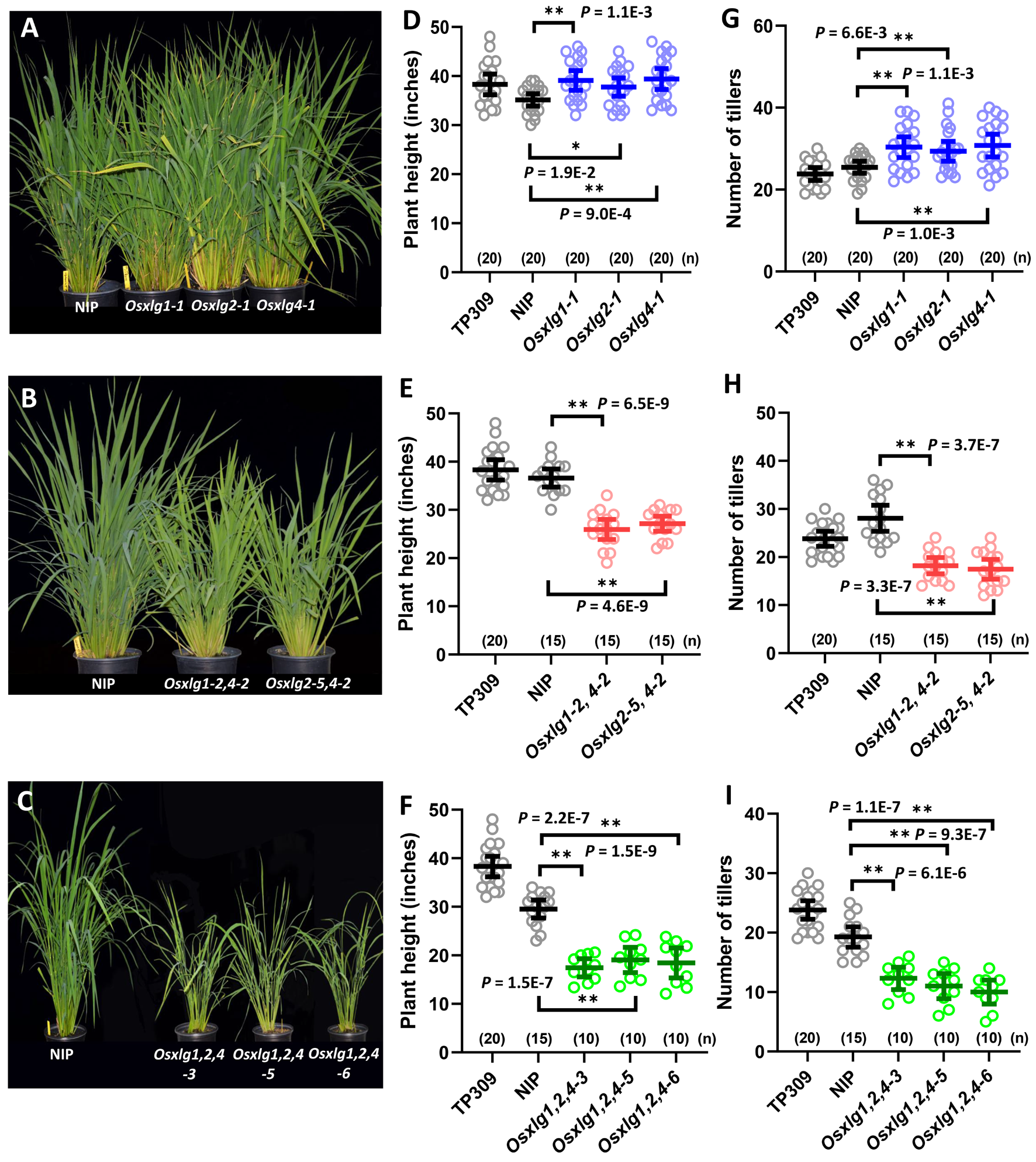

Supplement: Supplementary Figure 1 — Analysis of plant phenotype and development of Osxlg mutants at different time points. Growth phenotypes of 100-day-old rice WT NIP, Osxlg single (A), 90-day-old WT-NIP and double (B), and 70-day-old WT-NIP and triple mutant (C) plants. Ninety-five-day-old TP309 WT rice plant was used as an additional control in (D–I). The raw values for height and number of tillers of greenhouse grown WT rice and Osxlg mutants are shown in (D–I), respectively. The horizontal lines indicate the means, and the error bars represent 95% confidence intervals. n = sample size. Significance p-values are expressed as *p < 0.05, **p < 0.01. Significant differences between WT-NIP and Osxlg mutants were determined as calculated by statistical analysis using Statistica 5.0 with one-way ANOVA followed by Tukey’s multiple comparison test. [file Image_1.TIF]

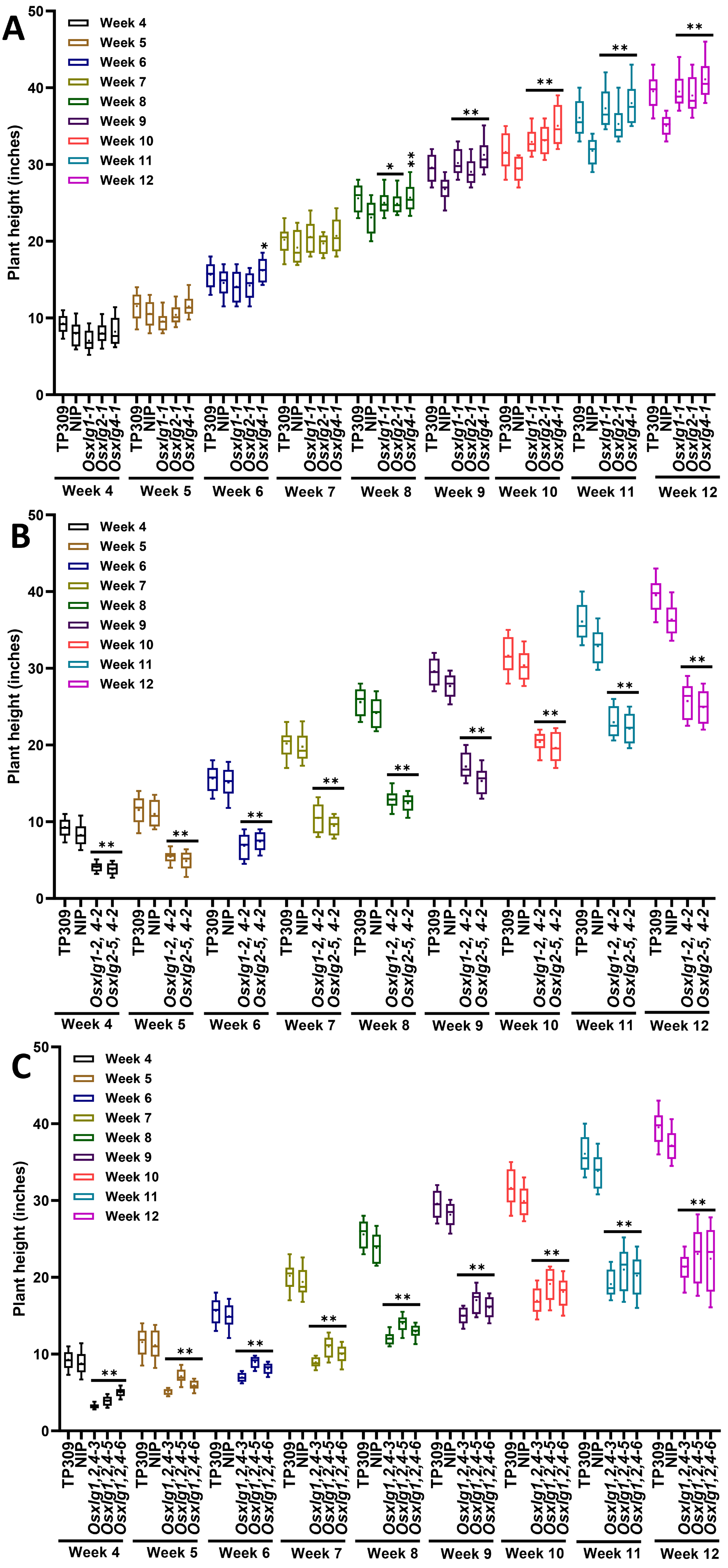

Supplement: Supplementary Figure 2 — Analysis of plant height of Osxlg mutants at different time points. Measurement of plant height (A–C) of Osxlg-KO mutants throughout the growth of the period from weeks 4 to 12 in comparison with the controls. Data are presented as box and whiskers plots showing the mean as “ + “ inside the boxes and the error bars represent 95% confidence intervals. For (A) n = 10 (TP309, NIP, Osxlg1), n = 12 (Osxlg-2 and Osxlg-4); For (B) n = 10 (TP309, NIP), n = 12 (Osxlg1-2, 4-2 and Osxlg 2-5, 4-2); For (C) n = 10 (TP309, NIP), n = 6 (Osxlg1,2,4-3; Osxlg1,2,4-5; Osxlg1,2,4-6). Significance p-values are expressed as *p < 0.05, **p < 0.01 by comparing WT-NIP with Osxlg mutants as calculated by statistical analysis using Statistica 5.0 with one-way ANOVA followed by Tukey’s multiple comparison test. [file Image_2.TIF]

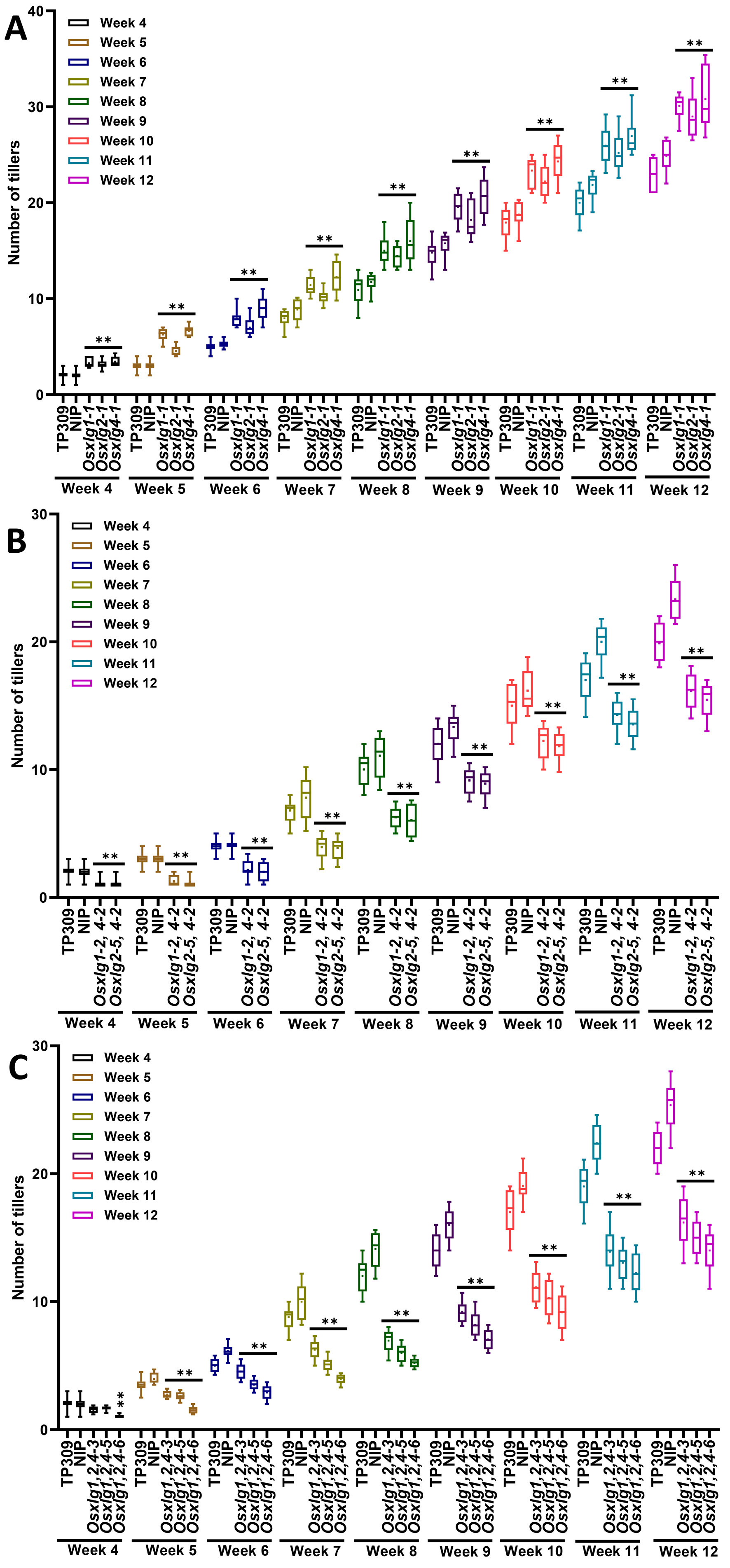

Supplement: Supplementary Figure 3 — Analysis of plant tiller growth and number of Osxlg mutants at different time points. Measurement of tiller numbers (A–C) of Osxlg-KO mutants throughout the growth of the period from weeks 4 to 12 in comparison with the controls. Data are presented as box and whiskers plots showing the mean as “ + “ inside the boxes and the error bars represent 95% confidence intervals. For (A) n = 10 (TP309, NIP, Osxlg1), n = 12 (Osxlg-2 and Osxlg-4); For (B) n = 10 (TP309, NIP), n = 12 (Osxlg1-2, 4-2 and Osxlg 2-5, 4-2); For (C) n = 10 (TP309, NIP), n = 6 (Osxlg1,2,4-3; Osxlg1,2,4-5; Osxlg1,2,4-6). p-Values are expressed as *p < 0.05, **p < 0.01. Significance differences between WT-NIP and Osxlg mutants were determined by statistical analysis using Statistica 5.0 with one-way ANOVA followed by Tukey’s multiple comparison test. [file Image_3.TIF]

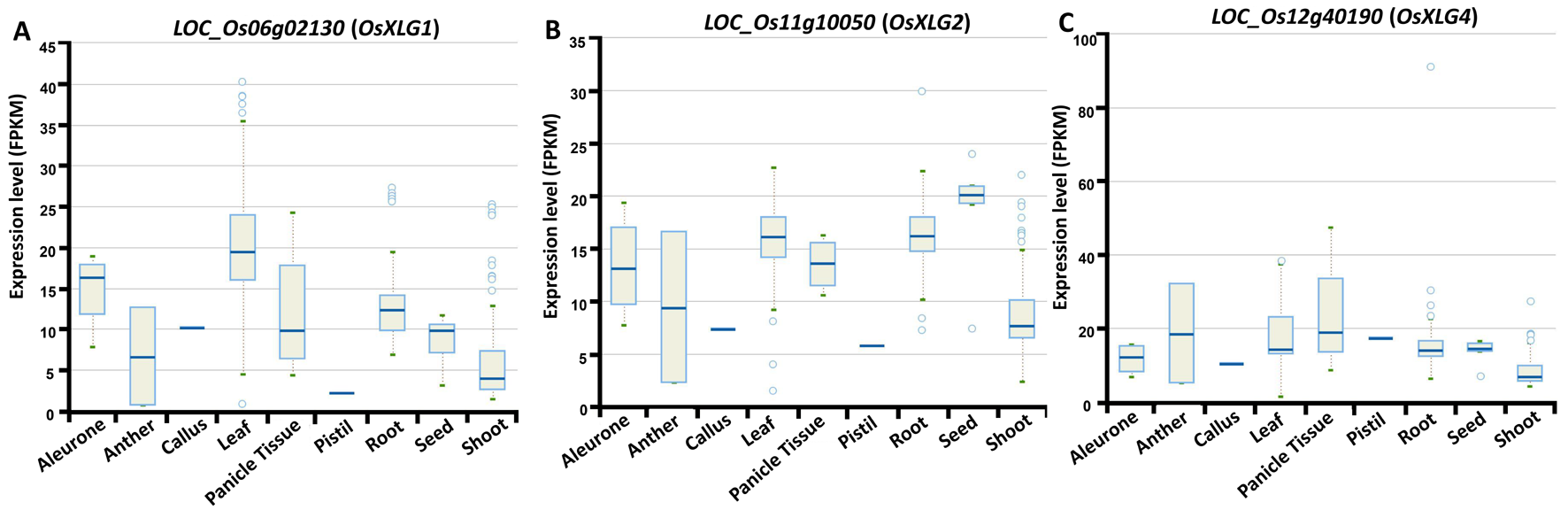

Supplement: Supplementary Figure 4 — Tissue-specific expression of XLGs in rice. Rice (A) OsXLG1 (LOC_Os06g02130), (B) OsXLG2 (LOC_Os11g10050), and (C) OsXLG4 (LOC_Os12g40190) expression in different tissues. The expression data were extracted from the meta-transcriptome data available at http://expression.ic4r.org/ (Xia et al., 2017). [file Image_4.TIF]
