# Supplementary material for: Novel Mutant Alleles Reveal a Role of the Extra-Large G Protein in Rice Grain Filling, Panicle Architecture, Plant Growth, and Disease Resistance
Source: Front Plant Sci. 2022 Jan 3;12:782960. doi: 10.3389/fpls.2021.782960 (PMC8761985; doi:10.3389/fpls.2021.782960)
Supplement: Supplementary file 5 [file Table_1.docx]

- **Supplementary Table 1:** Functions associated to plant G-protein complex

|  | **Function** | **Gene** | **Species** | **References** |
| --- | --- | --- | --- | --- |
| 1 | Cell proliferation | RGA1 | Rice | (Izawa et al., 2010) |
| 2 | Hormone response | RGA1 | Rice | (Ashikari et al., 1999; Oki et al., 2009) |
| 3 | Programmed cell death | RGA1 | Rice | (Steffens and Sauter, 2009) |
| 4 | Panicle architecture | GS3, DEP1 | Rice | (Fan et al., 2006; Takano-Kai et al., 2009; Zhou et al., 2009; Botella, 2012) |
| 5 | Grain size | Gβ, DEP1, GGC2 and GS3 | Rice | (Liu et al., 2018; Sun et al., 2018) |
| 6 | Plant growth/dwarf phenotype | RGA1 | Rice | Fujisawa et al., 1999 |
| 7 | Membrane transport / Ion channel homeostasis | Gα | Arabidopsis, *V. faba* tomato | (Armstrong and Blatt, 1995; Aharon et al., 1998; Wang et al., 2001; Jones and Assmann, 2004) |
| 8 | Pathogen resistance | RGA1 | Rice | (Suharsono et al., 2002; Komatsu et al., 2004; Assmann, 2005a; Liu et al., 2009) |
| 9 | Abiotic (drought, salinity, heat, and cold) stress tolerance | RGG1 and RGG2 | Rice | (Kato et al., 2004; Yadav et al., 2012; Colaneri et al., 2014; Urano et al., 2014; Ma et al., 2015; Jangam et al., 2016) |
| 10 | Nitrogen use efficiency | DEP1 | Rice | (Sun et al., 2014) |
| 11 | Seed germination | RGA1 | Rice | (Assmann, 2005b) |

**References**

Aharon, G. S., Gelli, A., Snedden, W. A., and Blumwald, E. (1998). Activation of a plant plasma membrane Ca2+ channel by TGα1, a heterotrimeric G protein α-subunit homologue. *FEBS Lett.* 424, 17–21. doi:10.1016/S0014-5793(98)00129-X.

Armstrong, F., and Blatt, M. R. (1995). Evidence for K+ channel control in Vicia guard cells coupled by G‐proteins to a 7TMS receptor mimetic. *Plant J.* 8, 187–198. doi:10.1046/j.1365-313X.1995.08020187.x.

Ashikari, M., Wu, J., Yano, M., Sasaki, T., and Yoshimura, A. (1999). Rice gibberellin-insensitive dwarf mutant gene Dwarf 1 encodes the alpha-subunit of GTP-binding protein. *Proc. Natl. Acad. Sci. U. S. A.* 96, 10284–9. doi:10.1073/PNAS.96.18.10284.

Assmann, S. M. (2005a). G Protein Regulation of Disease Resistance During Infection of Rice with Rice Blast Fungus. *Sci. Signal.* 2005, cm13–cm13. doi:10.1126/stke.3102005cm13.

Assmann, S. M. (2005b). G Protein Signaling in the Regulation of Rice Seed Germination. *Sci. Signal.* 2005, cm12–cm12. doi:10.1126/stke.3102005cm12.

Botella, J. R. (2012). Can heterotrimeric G proteins help to feed the world? *Trends Plant Sci.* 17, 563–568. doi:10.1016/j.tplants.2012.06.002.

Colaneri, A. C., Tunc-Ozdemir, M., Huang, J., and Jones, A. M. (2014). Growth attenuation under saline stress is mediated by the heterotrimeric G protein complex. *BMC Plant Biol.* 14, 129. doi:10.1186/1471-2229-14-129.

Fan, C., Xing, Y., Mao, H., Lu, T., Han, B., Xu, C., et al. (2006). GS3, a major QTL for grain length and weight and minor QTL for grain width and thickness in rice, encodes a putative transmembrane protein. *Theor. Appl. Genet.* 112, 1164–1171. doi:10.1007/s00122-006-0218-1.

Fujisawa, Y., Kato, T., Ohki, S., Ishikawa, A., Kitano, H., Sasaki, T., et al. (1999). Suppression of the heterotrimeric G protein causes abnormal morphology, including dwarfism, in rice. *Proc. Natl. Acad. Sci. U. S. A.* 96, 7575–80. doi:10.1073/pnas.96.13.7575.

Izawa, Y., Takayanagi, Y., Inaba, N., Abe, Y., Minami, M., Fujisawa, Y., et al. (2010). Function and expression pattern of the alpha subunit of the heterotrimeric G protein in rice. *Plant Cell Physiol.* 51, 271–281. doi:10.1093/pcp/pcp186.

Jangam, A. P., Pathak, R. R., and Raghuram, N. (2016). Microarray Analysis of Rice d1 (RGA1) Mutant Reveals the Potential Role of G-Protein Alpha Subunit in Regulating Multiple Abiotic Stresses Such as Drought, Salinity, Heat, and Cold. *Front. Plant Sci.* 7, 1–15. doi:10.3389/fpls.2016.00011.

Jones, A. M., and Assmann, S. M. (2004). Plants: The latest model system for G-protein research. *EMBO Rep.* 5, 572–578. doi:10.1038/sj.embor.7400174.

Kato, C., Mizutani, T., Tamaki, H., Kumagai, H., Kamiya, T., Hirobe, A., et al. (2004). Characterization of heterotrimeric G protein complexes in rice plasma membrane. *Plant J.* 38, 320–31. doi:10.1111/j.1365-313X.2004.02046.x.

Komatsu, S., Yang, G., Hayashi, N., Kaku, H., Umemura, K., and Iwasaki, Y. (2004). Alterations by a defect in a rice G protein α subunit in probenazole and pathogen-induced responses. *Plant, Cell Environ.* 27, 947–957. doi:10.1111/j.1365-3040.2004.01202.x.

Liu, H., Ramanujam, R., and Naqvi, N. I. (2009). “Surface Sensing and Signaling During Initiation of Rice-Blast Disease,” in *Advances in Genetics, Genomics and Control of Rice Blast Disease*, eds. G. Wang and B. Valent (Dordrecht: Springer Netherlands), 23–32. doi:10.1007/978-1-4020-9500-9_3.

Liu, Q., Han, R., Wu, K., Zhang, J., Ye, Y., Wang, S., et al. (2018). G-protein βγ subunits determine grain size through interaction with MADS-domain transcription factors in rice. *Nat. Commun.* 9, 852. doi:10.1038/s41467-018-03047-9.

Ma, Y., Dai, X., Xu, Y., Luo, W., Zheng, X., Zeng, D., et al. (2015). COLD1 confers chilling tolerance in rice. *Cell* 160, 1209–1221. doi:10.1016/j.cell.2015.01.046.

Oki, K., Inaba, N., Kitagawa, K., Fujioka, S., Kitano, H., Fujisawa, Y., et al. (2009). Function of the α subunit of rice heterotrimeric G protein in brassinosteroid signaling. *Plant Cell Physiol.* 50, 161–172. doi:10.1093/pcp/pcn182.

Steffens, B., and Sauter, M. (2009). Heterotrimeric G protein signaling is required for epidermal cell death in rice. *Plant Physiol.* 151, 732–40. doi:10.1104/pp.109.142133.

Suharsono, U., Fujisawa, Y., Kawasaki, T., Iwasaki, Y., Satoh, H., and Shimamoto, K. (2002). The heterotrimeric G protein subunit acts upstream of the small GTPase Rac in disease resistance of rice. *Proc. Natl. Acad. Sci.* 99, 13307–13312. doi:10.1073/pnas.192244099.

Sun, H., Qian, Q., Wu, K., Luo, J., Wang, S., Zhang, C., et al. (2014). Heterotrimeric G proteins regulate nitrogen-use efficiency in rice. *Nat. Genet.* 46, 652–656. doi:10.1038/ng.2958.

Sun, S., Wang, L., Mao, H., Shao, L., Li, X., Xiao, J., et al. (2018). A G-protein pathway determines grain size in rice. *Nat. Commun.* 9. doi:10.1038/s41467-018-03141-y.

Takano-Kai, N., Hui, J., Kubo, T., Sweeney, M., Matsumoto, T., Kanamori, H., et al. (2009). Evolutionary history of GS3, a gene conferring grain length in rice. *Genetics* 182, 1323–1334. doi:10.1534/genetics.109.103002.

Urano, D., Colaneri, A. C., and Jones, A. M. (2014). Gα modulates salt-induced cellular senescence and cell division in rice and maize. *J. Exp. Bot.* 65, 6553–6561. doi:10.1093/jxb/eru372.

Wang, X. Q., Ullah, H., Jones, A. M., and Assmann, S. M. (2001). G protein regulation of ion channels and abscisic acid signaling in Arabidopsis guard cells. *Science* 292, 2070–2. doi:10.1126/science.1059046.

Yadav, D. K., Islam, S. M. S., and Tuteja, N. (2012). Rice heterotrimeric G-protein gamma subunits (RGG1 and RGG2) are differentially regulated under abiotic stress. *Plant Signal. Behav.* 7, 733–740. doi:10.4161/psb.20356.

Zhou, Y., Zhu, J., Li, Z., Yi, C., Liu, J., Zhang, H., et al. (2009). Deletion in a quantitative trait gene qPE9-1 associated with panicle erectness improves plant architecture during rice domestication. *Genetics* 183, 315–324. doi:10.1534/genetics.109.102681.
