# Supplementary material for: Novel Mutant Alleles Reveal a Role of the Extra-Large G Protein in Rice Grain Filling, Panicle Architecture, Plant Growth, and Disease Resistance
Source: Front Plant Sci. 2022 Jan 3;12:782960. doi: 10.3389/fpls.2021.782960 (PMC8761985; doi:10.3389/fpls.2021.782960)
Supplement: Supplementary file 6 [file Table_2.DOCX]

**Supplementary Table 2:** List of primer sequences used

| Sl. | Primer name | Sequence (5’-3’) | Target plants |
| --- | --- | --- | --- |
| 1 | OsUbi_1051F | tcatgcctgagtgattggtgc | All XLG transgenic lines |
| 2 | Cas9_868R | gaaacaggtcggcgtactggtc | All XLG transgenic lines |
| 3 | OsXLG1_412F | ggattcctcaatccgcggtgaa | OsXLG1 |
| 4 | OsXLG1_1224R | gtagctgcaaaacctcgcgtc | OsXLG1 |
| 5 | OsXLG2_105F | atttcgcagccgcgggtttgga | OsXLG2 |
| 6 | OsXLG2_547R | gcaatccgggacactgggagc | OsXLG2 |
| 7 | OsXLG4_473F | cgctgttgaagtcccaccacc | OsXLG4 |
| 8 | OsXLG4_1035R | ccggagcttagaaggagggcac | OsXLG4 |
