# Supplementary material for: Novel Mutant Alleles Reveal a Role of the Extra-Large G Protein in Rice Grain Filling, Panicle Architecture, Plant Growth, and Disease Resistance
Source: Front Plant Sci. 2022 Jan 3;12:782960. doi: 10.3389/fpls.2021.782960 (PMC8761985; doi:10.3389/fpls.2021.782960)
Supplement: Supplementary file 7 [file Table_3.docx]

**Supplementary Table 3:** Novel *Osxlg* alleles

| **Allele** | **Indel or substitution** | **Nonsense or missense Mutation** |
| --- | --- | --- |
| *Osxlg1*-3 | Deletion of 32 bases. | Frameshift mutation leading to a stop codon at residue 111. |
| *Osxlg1*-4 | Deletion of 3 bases (GAT) around the target sequence. | Deletion of one amino acid (I) at residue 61. |
| *Osxlg1*-5 | 3 base (ATT) deletions at the target site. | Deletion of a single amino acid. |
| *Osxlg1*-6 | Deletion of 18 bases. | Deletion of 6 amino acids at the same location. |
| *Osxlg2*-2 | Single base deletion. | Frameshift mutation from residue 37 and a STOP codon at residue 152. |
| *Osxlg2*-4 | 2 base substitution in the target site. | Substitution one amino acid at residue 38. |
| *Osxlg2*-6 | One base insertion at the target site. | This generated an immediate STOP codon. |
| *Osxlg2*-7 | One base was deleted at the target site. | Frameshift mutation from residue 38 and introduces a STOP codon at residue 151. |
| *Osxlg2*-9 | 4 base deletions at the target site. | Frameshift mutation leading to a STOP codon at residue 152. |
| *Osxlg4*-4 | 2 base deletion. | Frameshift mutation from residue 270 leading to a STOP codon at residue 343. Similar to *Osxlg4*-3 |
| *Osxlg4*-5 | Single base insertion. | Frameshift mutation leading to a STOP codon at residue 342. |
| *Osxlg4*-6 | 2 base deletion. | Frameshift mutation leading to a STOP codon at residue 342. Similar to *Osxlg4*-5. |
