# Supplementary material for: Novel Mutant Alleles Reveal a Role of the Extra-Large G Protein in Rice Grain Filling, Panicle Architecture, Plant Growth, and Disease Resistance
Source: Front Plant Sci. 2022 Jan 3;12:782960. doi: 10.3389/fpls.2021.782960 (PMC8761985; doi:10.3389/fpls.2021.782960)
Supplement: Supplementary file 8 [file Table_4.DOCX]

**Supplementary Table 4:** Comparison of agronomic traits on *Osxlg* mutants. All data except grain length rounded to nearest integer. Values in bold font indicate mutant traits significantly different from WT Nipponbare (higher - black font, lower – dark red font) from WT values for NSU and UGA’s GH data. na: not analyzed. A dash “- “and plus “+” designate that the mutant’s lines that are significantly decreased or increased compared to WT respectively for Cui et al. (2020).

| Plant Types | Plant height (inches) | Number of tillers | Dry biomass yield (g/plant) | Seed setting rate (%) | Days to heading (d) | Panicle number | Panicle length (cm) | Grain number per panicle | Total panicle weight (g/plant) | Grain length per 10 seeds (cm) | Seed dry weight  (mg/10 seeds)/(1000 grain weight, g) |
| --- | --- | --- | --- | --- | --- | --- | --- | --- | --- | --- | --- |
| **Cui et al. BMC Plant Biology (2020) 20: 90** | | | | | | | | | | | |
| WT (Sasanishiki) |  |  |  |  |  |  |  |  |  |  |  |
| *Psxlg1-1/1-2* | **-** |  | na | ns | **-** | **+** | **-** | **+** | na | **+** | **+** |
| *Psxlg2-1/2-1* | ns |  | na | **-** | **-** | ns | ns | ns | na | ns | **-** |
| *Psxlg3-1/3-2* | **-** |  | na | **-** | **-** | **+** | ns | ns | na | ns | ns |
| *Psxlg4-1/4-2* | ns |  | na | ns | **-** | ns | ns | **-** | na | ns | ns |
| **National University of Singapore (NUS) GH growth data** | | | | | | | | | | | |
| WT (Nipponbare) | 29 | 5 | na | 68 | na | na | 18 | na | na | 3.7 | 240 |
| *Osxlg1-1* | **26** | 6 | na | **18** | na | na | **15** | na | na | **3.6** | **210** |
| *Osxlg2-1* | **27** | 6 | na | **51** | na | na | **16** | na | na | **3.8** | 240 |
| *Osxlg4-1* | **28** | 6 | na | 66 | na | na | 18 | na | na | 3.8 | 240 |
| *Osxlg1-2, 4-2* | **24** | **4** | na | **5** | na | na | **14** | na | na | **3.5** | **200** |
| **University of Georgia (UGA) GH growth data** | | | | | | | | | | | |
| WT (Nipponbare) | 35 | 25 | 201 | 82 | 116 | 72 | 19 | 128 | 162 | 7.9 | 246 |
| *Osxlg1-1* | **39** | **30** | **213** | **57** | **106** | **47** | **15** | **79** | **113** | **7.3** | **197** |
| *Osxlg2-1* | **38** | **29** | **216** | **70** | **106** | **63** | **16** | **76** | **122** | **8.4** | **215** |
| *Osxlg4-1* | **39** | **30** | **218** | **77** | **108** | 69 | **17** | **104** | **131** | **8.5** | **219** |
| *Osxlg1-2, 4-2* | **24** | **17** | **163** | **49** | **103** | **52** | **15** | **89** | **109** | **6.5** | **185** |
| *Osxlg2-5, 4-2* | **25** | **16** | **169** | **53** | **105** | **59** | **16** | **94** | **101** | **6.9** | **191** |
| *Osxlg1,2,4-3* | **21** | **15** | **155** | **46** | **101** | **57** | **18** | **107** | **98** | **6.6** | **171** |
| *Osxlg1,2,4-5* | **23** | **16** | **161** | **45** | **102** | **53** | **17** | **105** | **80** | **6.4** | **154** |
| *Osxlg1,2,4-6* | **22** | **14** | **163** | **43** | **104** | **55** | **17** | **94** | **90** | **6.8** | **172** |
